# Evaluating item difficulty patterns for assessing student misconceptions in science across physics, chemistry, and biology concepts

**DOI:** 10.1016/j.heliyon.2021.e08352

**Published:** 2021-11-12

**Authors:** Soeharto Soeharto, Benő Csapó

**Affiliations:** a Doctoral School of Education, University of Szeged, 32-34, Petőﬁ S. sgt., Szeged, H-6722, Hungary; b Institute of Education, University of Szeged, Hungary; c MTA-SZTE Research Group on the Development of Competencies, Hungary

**Keywords:** Item difficulty, Rasch measurement, Misconception, Science, Diagnostic test

## Abstract

Understanding item difficulty in science concepts is essential for teachers in teaching and learning to avoid student misconceptions. This study aims to evaluate the patterns of item difficulty estimates in science concepts exploring student misconceptions across physics, biology, and chemistry and to explore differential item functioning (DIF) items in the developed diagnostic test on the basis of gender and grade. Participants were drawn from 856 students (52.3% females and 47.7% males) comprising senior high school students from 11^th^ to 12^th^ grades and pre-service science teachers in the West Kalimantan province, Indonesia. Out of 16 science concepts categorized, the common science concepts causing misconceptions among students were investigated to understand item difficulty patterns using Rasch measurement. The findings of this study evaluated that 32 developed items are valid and reliable whereby the item difficulty estimates ranged from −5.13 logits to 5.06 logits. Chemistry is the scientific discipline with the highest mean logits than other disciplines. There is no significant item difficulty estimate across the science disciplines. We also found DIF issues in one item based on gender and four items based on grade. This study contributes a significant role in mapping and informing item difficulty patterns in science concepts to tackle teachers' problems in assessing and teaching science concepts to improve the students’ science performance. Future studies and limitations are also discussed.

## 1 Introduction

Science concepts are critical elements in explaining and understanding natural phenomena across all science disciplines. The particular science concept provides a practical framework for integrating science disciplines and has a significant impact on the learning process and in the thinking and modeling of natural and technological processes. Several studies reported students' difficulties in learning scientific concepts. Soeharto (2017) reported that students suffered misconceptions about impulses and momentum because of a lack of understanding of various types of collision. Additionally, Tiruneh et al. (2017) found that students experienced difficulty in solving critical thinking problems related to electricity and magnetism. Students' weaknesses in understanding science concepts across science disciplines are attributed to how some science concepts are introduced and applied in varied ways that are often incompatible (Cooper and Klymkowsky, 2013; Lancor, 2014). Several concepts in science are complex for students to understand, causing them to experience misconceptions. Wandersee et al. (1994) analyzed 103 studies related to misconceptions in the paradigm of science concepts, Gurel et al. (2015) found 273 articles about misconceptions across science disciplines and instruments to assess students’ understanding, and Soeharto et al. (2019) found 111 articles from 2015 to 2019 that focused on student misconceptions across the disciplines of physics, biology, and chemistry. Understanding the science concepts properly will help students to work on problems of varying degrees of difficulty. Thus, the investigation of difficulty levels of science concepts across science disciplines has the potential to hamper students through suffering misconceptions and thereby failing to achieve their best performance in science.

Indonesian students' science performances were ranked the lowest in the 2018 PISA report involving 41 countries as target participants (OECD, 2020). The issue of difficulties in understanding science concepts across science disciplines should be addressed to improve student learning outcomes. Several studies have been conducted to investigate conceptions in various science concepts across various disciplines (e.g., Butler et al. (2015), Korur (2015), Park and Liu (2019), Peterson et al. (1989), Tiruneh et al. (2017); Tümay (2016)). However, comparing the actual difficulty level of science concepts from various disciplines becomes a problem and is challenging to implement. The results of an instrument test may reflect differences in the respondents’ abilities and the lack of ability to work on questions in various science disciplines. Hence, there is a necessity to create an instrument that allows for a standardized measurement of science concepts in various scientific fields so that teachers can recognize the especially challenging science concepts whenever they teach students in various areas of science.

The goal of objective measurement locates at the core of science, and science education research should also attempt to carry the instrument according to objective measurement criteria. Our study evaluates item difficulty estimates using a standardized instrument to assess the distributed science concepts misconceptions to students across the science disciplines using Rasch measurement and explores DIF. Although some research concentrates on students' science conceptions of particular concepts, to what extent students have experienced the ease or difficulty in understanding science concepts has not been fully elucidated using a standardized instrument to measure concepts comprehension across science disciplines. This study will fill the gap in empirical research that provides evidence related to students’ difficulties in understanding science concepts across disciplines, especially science concepts that generate misconceptions in students on the basis of key concepts in the findings of previous research findings by Soeharto et al. (2019). Previous studies on pre-service science teachers and undergraduate students are limited (Singer, 2013), and some studies focus more on students at the secondary school level (Erman, 2017; Slater et al., 2018; Tiruneh et al., 2017; Tümay, 2016). This study will target both groups, students at secondary school and teachers who have completed pre-service and are undergoing education based on the Indonesian science core curriculum.

## 2 Literature review

### 2.1 Student misconceptions of science concepts

Misconceptions are defined as misunderstandings and interpretations that are not scientifically accurate, showing inaccurate prior insight and wrong ideas (Cooper and Klymkowsky, 2013; Ebert-May et al., 2004; Van Den Broek and Kendeou, 2008). Misconceptions come from various sources; students, teachers, textbooks, and the wider environment (Van Den Broek and Kendeou, 2008). In formal education, scientific misconceptions have been found through interactions between teachers and students who may experience misconceptions in the learning process. Student misconceptions are difficult to identify using traditional methods. Teachers must understand students’ misconceptions in learning and increase their correct conceptions (Brehm et al., 1986). Many scientific concepts are difficult to understand, which causes students to generate misconceptions (Gurel et al., 2015; Soeharto et al., 2019). Educators who teach science concepts with certain strategies may, without realizing it, actually strengthen and spread misconceptions. Thus, educators must understand the level of difficulty of scientific concepts and which precise concepts cause misconceptions in students (Burgoon et al., 2011).

Numerous studies have been conducted regarding students; the understanding of science concepts in various disciplines (e.g., Laliyo et al., 2020; Liampa et al., 2019; Mubarokah et al., 2018; Planinic et al., 2019; Prodjosantoso et al., 2019). Students in secondary school held misconceptions in physics and were finding it challenging to distinguish between the concepts of wave, energy, impulse, and momentum (Caleon and Subramaniam, 2010; Kaltakci-Gurel et al., 2017; Korur, 2015; Soeharto, 2017; Taslidere, 2016). Undergraduate students also suffered similar difficulties in distinguishing the concepts of astronomy and geometrical optics (Kaltakci-Gurel et al., 2017; Slater et al., 2018). Ding et al. (2013) found that students misunderstood the concept of light in an energy context because they had experienced misconceptions in traditional physics learning in the classroom. In chemistry, undergraduate students had identified difficulties in understanding the relationship between molecular bonds and energy (Becker and Cooper, 2014). Additionally, pre-service science teachers mostly conceptualize heat as a material without evaluating its size or grade (Lewis and Linn, 1994). In biology, students fail to explain feeding relationships as a means of energy transfer in food chains (Wernecke et al., 2018). Galvin and Mooney (2015) also found that student misconception was caused by mistakes in the biology class at the secondary school and college student levels. Chabalengula et al. (2012) also investigated first-year college students concerning their understanding of the concept of science in biology and found that students failed to understand and apply energy concepts to the human body system and feeding relationships to explain life processes using aspects of energy transformation.

Although most research is related to student misconceptions in science concepts across disciplines, only a few studies focus on understanding the inherent difficulty level of items in science concepts in various science disciplines (e.g., Liu et al. (2015); Park and Liu (2019)). Recently, Lancor (2015) and Chen et al. (2014) found that students' understanding of science concepts is different for each discipline, which implied the importance of understanding the difficulty level of items in science concepts across science disciplines. Students must be able to develop their understanding of scientific concepts across all disciplines to achieve the success of the learning objectives (Krajcik et al., 2014). This finding proves that the level of difficulty in scientific concepts will be able to hinder the development of students' understanding in learning. Knowing science concepts embedded in various disciplines is necessary to investigate students’ strengths and weaknesses against different scientific concepts so that teachers can have the empirical evidence required to teach science concepts across the science disciplines better.

### 2.2 Instruments for assessing student misconceptions

Student misconceptions are difficult to identify with traditional methods. Educators have to revise and identify student misconceptions to help students understand new concepts and finally provide opportunities for students to apply these concepts to science problems (Butler et al., 2015). To evaluate and identify students' basic knowledge of concepts in science, researchers used a diagnostic test. The diagnostic test assesses students' proportional knowledge on the basis of the science content, the science teacher can develop a clear idea about the nature of the students’ knowledge by using a diagnostic test at the beginning or the end of the learning activity (Peterson et al., 1989; Taslidere, 2016; Treagust, 1986).

Researchers in science majors have used and developed numerous instruments to assess student misconceptions or student conceptual understanding (Soeharto et al., 2019). Two-tier multiple-choice diagnostic tests are the most reliable assessment tool developed to identify student misconceptions in science education majors because the multiple-choice test merely assessed student content knowledge without considering the reasoning behind students' responses (Chabalengula et al., 2012; Gurel et al., 2015; Soeharto et al., 2019). In a two-tier multiple-choice test, the first tier assesses students' insight about science concepts, whereas the second tier investigates student reasoning for their choices in the first tier. However, the two-tier multiple-choice test cannot differentiate students' mistakes due to lack of knowledge or simply guessing answers (Caleon and Subramaniam, 2010; Chabalengula et al., 2012). Thus, scholars introduced having the Certainty Response Index (CRI) embedded in the question, which measures the respondent level certainty in the first two tiers, and they call this test the three-tier multiple-choice diagnostic test (Gurcay and Gulbas, 2015; Peşman and Eryılmaz, 2010). However, regardless of the students having right or wrong answers, the answers with a low level of confidence were categorized as a lack of knowledge, and wrong answers with a high level of confidence were categorized as a misconception (Kaltakci-Gurel et al., 2017; Peşman and Eryılmaz, 2010). Instead, of using the confidence level choices or CRI on a three-tier or four-tier multiple-choice diagnostic test to differentiate between students’ guessed answers or lack of knowledge answers, this study tries a new approach to analyze items: two-tier multiple diagnostic tests using an objective instrument based on Rasch measurement. The Rasch measurement was chosen because this analysis can provide accurate results of the level of student ability and the difficulty of items, even analyzing the likelihood of students just guessing the answers (Sumintono and Widhiarso, 2014).

### 2.3 Rasch measurement

Rasch measurement is a measurement model developed by the Danish mathematician, George Rasch. Rasch measurement is formed on the basis of item–person interactions and probability estimates. Using equations, the interaction between the item and person can be elucidated and described. People who have low ability should not de facto be able to answer items that have a high difficulty level (Andrich, 2018). The probability in Rasch measurement is determined based on the item difficulty level and the person's ability simultaneously. Moreover, the probability of answering items is differentiated by item difficulty level and individual ability (Boone et al., 2013; Khine, 2020; Planinic et al., 2019). Item difficulty level and person ability are generated and determined based on a log odds unit scale (logits) as interval data, thereby ensuring that person and item parameters are entirely independent (Bond et al., 2020; Sumintono and Widhiarso, 2014). In other words, a person's ability in a measurement remains the same regardless of the item difficulty level, and the item difficulty level does not change regardless of the person's ability. For dichotomous model, the mathematical derivation of the Rasch analysis is:

$$log\frac{P_{ni1}}{P_{ni0}}=B_{n}-D_{i}$$

where.

$P_{ni1}$ or $P_{ni0}$ is the probability that person n encountering item i is observed in category 1 or 0,

$B_{n}$ is the "ability" (theta) measure of person n,

$D_{i}$ is the "difficulty" (delta) measure of item i, the point where the highest and lowest categories of the item are equally probable.

(Linacre‬, 2021b)‬‬‬‬‬

This study focused on analyzing item difficulty levels for science concepts across disciplines using two-tier multiple-choice diagnostic tests. The Rasch dichotomous model was used to analyze dichotomy data, where 0 was categorized as the misconception and 1 was categorized as the correct answer. The dichotomy data were used to generate the item difficulty level in logits. Rasch measurement was chosen because this method can overcome some limitations in Classical Test Theory (CTT) such as (a) the measurement or data analysis was constructed using interval data not categorical or nominal data; (b) the items' difficulty level and the person's ability are independent; (c) the parameter reliability can measure items and persons and depends on the size of the sample; and (d) the data on the measurement of Rasch explains the response at the individual level, not group-centred statistics (Barbic and Cano, 2016).

## 3 Research questions

The study investigates item difficulty patterns, item–person map interaction, and DIF based on gender and grade across science disciplines using the two-tier multiple-choice diagnostic test for assessing student misconceptions. Hence, we set out the following research questions;

(1) Are the items on the instrument used valid and reliable?
(2) What are the item difficulty patterns measured by diagnostic instruments for assessing student misconceptions on science concepts?
(3) To what extent are the item difficulties able to describe the concepts that cause students misconceptions across disciplines and science concepts?
(4) Are there any DIF issues based on gender and grade?

## 4 Methods

### 4.1 Participants

Participants were drawn from 856 senior high school students and a pre-service science teacher in Pontianak, West Kalimantan province, Indonesia. We selected 11 classes randomly from five different schools in total as representative schools in this area. All participants in this study were students from three different school levels, 10th, 11th, and 12th grades, and pre-service science teachers. The paper-based test was administered at the schools and university. Students and pre-service science teacher spent 120 min completing the test under the supervision of researchers and teachers. Table 1 presents the demographic characteristics of the participants.

**Table 1:** Demographic characteristics of participants in this study.

Demographic characteristics		Frequency	Percentage (%)
Gender	Females	448	52.3
	Males	408	47.7
Grade	10th	231	27.0
	11th	291	34.0
	12th	153	17.9
	Pre-service science teacher (PST)	181	21.1
School category	Public	621	72.5
	Private	235	27.5
Living place	City	444	51.9
	District	412	48.1

### 4.2 Instruments

#### 4.2.1 Background questionnaires

The background questionnaire was adapted from the Indonesian version of the PISA 2015 SES instrument (OECD, 2016). The questionnaire is embedded in the developed diagnostic test body. The background questionnaire in this study consists of information such as gender, grade, school category, home address, parents' education, and parents' jobs. However, we omit the parents' education, home address, school category, and job data because we want to analyze item difficulties' patterns across science disciplines and analyze item differences’ function on the basis of grade and gender.

#### 4.2.2 The development of the two-tier multiple-choice diagnostic test

To capture student misconceptions or alternative conceptions, we implemented the developed two-tier multiple-choice diagnostic test. The two-tier test cannot differentiate students who are just guessing answers and related confidence level, and some researchers usually applied CTT analysis and the CRI (Hasan et al., 1999). Otherwise, Rasch measurement can overcome the weakness of two-tier tests with CTT and CRI analysis in cases of the certainty level and can provide a comprehensive and objective measure (Barbic and Cano, 2016). Before constructing and developing the instrument, the researcher investigated some literature review studies and misconceptions in science handbooks (AAAS, 2012; Allen, 2014; Csapó, 1998; Soeharto et al., 2019). This process was conducted to find common rationales behind misconceptions in science. Sixteen concepts were selected and adjusted to the Indonesian education curriculum for Curriculum 2013, especially on the senior high school level from the physics, biology, and chemistry concepts represented in Table 2. Thirty-two item questions were adapted developed in the form of a two-tier multiple-choice diagnostic test with eight items is adapted from the American Association for the Advancement of Science (AAAS) (2012), two items adapted from (Csapó, 1998), 23 items newly designed by authors. The backward‒forward translation process from English to Indonesian was conducted by two science and mathematics instructors and researchers. Table 3 represents a sample item from the force concept.

**Table 2:** Concepts and item number in the developed two-tier multiple-choice diagnostic test.

Subject	Concept	Item numbers	Total item
Physics	Kinetic energy, thermodynamics–thermal energy, atoms and molecules, impulse and momentums, light, and force	1, 2, 3, 4, 5, 6, 7, 8, 9,10, 11, 12	12
Biology	Human body systems, cells, breathing, feeding relationships, microbes, and disease	13, 14, 15, 16, 17, 18, 19, 20, 21, 22	10
Chemistry	Chemical compounds, substances and chemical reactions, redox reaction, hydrocarbons, and chemicals equilibrium	23, 24, 25, 26, 27, 28, 29, 30, 31, 32	10

**Table 3:** Sample item of the two-tier multiple-choice diagnostic test on the force concept.

First tier	The book with a weight of 10 N is placed on the table as shown. The book is in rest condition. Which of the following statements is correct? a) The book at rest condition has no forces acting upon it. b) The book has a weight force of 10 N and a reaction force of 10 N. c) The book has a contact force of 10 N. d) The book has weight force, contact force, and reaction force with equal quantity; each force has 10 N.
Second tier	Which one of the following is the reason for your answer to the previous question? a) When the resultant forces in the book are zero, no forces work on the book. b) All forces in the book have equal quantity, but the resultant forces in the book are not zero because the book holds on to the table. c) The resultant forces in the book are the same as the number of all forces working on the book's system. d) When the book is in a rest condition, all forces negate each other. e) ….….….….….….….….….….….….….….….….….….….….….….….….….….….…….

The two-tier multiple-choice diagnostic test consists of two-level questions. The first tier question asks about science content, and the second tier question asks about scientific reasoning. Students can choose one choice in the second tier or write down their own reason in the form of an open-ended answer to explain the related science content. Peterson et al. (1989) supported this two-tier test format since most multiple-choice questions did not provide sufficient information to explain the students' reasoning, whereas the additional explanation items in second-tier questions can assess students’ understanding related to science concepts and diagnose misconceptions.

### 4.3 Procedures, scoring, and data analysis

Before applying data collection in schools and universities, researchers asked permission to administer the tests to related institutions and were granted ethical research approval from the university. With the help and supervision of teachers, the paper-based test was implemented in the classroom. For item scoring, the correct answer was scored as 1 point, and an incorrect answer was scored as 0 points for all the items. Students get 1 point if they address the task correctly in the first and second tiers.

The Winsteps version 4.8.0 software (Linacre‬, 2021a) and Statistical Package for the Social Sciences (SPSS) version 25 (IBM SPSS, 2017) were applied in this study. Rasch analysis and some statistical methods such as descriptive statistics, internal consistency using Cronbach alpha were performed in data analysis. All samples in the data set were investigated and included in the data analysis. Winsteps software ran the analysis based on joint maximum likelihood estimation equations; in this formulation, we produced item difficulty scores (IFILE) in log odds unit scale (logits) from student raw scores. Logits are interval data ranging from a specific value from negative infinity to a positive infinity number (Linacre, 1998, 2020). Item difficulty data in logits will be used as a data variable to evaluate reliability, validity, the item difficulty pattern, and DIF using Rasch analysis. Rasch analysis has some advantages in explaining the psychometric properties of data such as (1) generating the difficulty level of an item accurately and precisely, (2) detecting the suitability and interaction of items and persons (item–person maps), (3) identifying outliers (person misfit), and (4) detecting item bias (DIF), which is useful for exploring item difficulties’ patterns in this study (Boone et al., 2016; Sumintono and Widhiarso, 2014).‬‬‬‬‬‬‬‬‬‬‬‬‬‬‬‬‬‬‬‬‬‬‬‬‬‬‬‬‬‬‬‬‬‬‬‬‬

## 5 Results

### 5.1 Reliability and validity

Rasch analysis provided two parameters of reliability; item reliability and person reliability, ranging from 0 to 1. Both the item and person reliability are acceptable in this study at 1.00 and 0.8, respectively (Fisher, 2007), and the item internal consistency using Cronbach's alpha value for all items is 0.88 (Taber, 2018). Item reliability is considered excellent if the value is close to 1 (Fisher, 2007; Sumintono and Widhiarso, 2014). It is possible to achieve if a stable item measure is used for measuring stable person measure above 500, the minimum criteria are 30 items for measuring 30 participants that can generate statistically stable measures with 95 % confidence and ±1.0 logits (Azizan et al., 2020). These results establish that the instrument used is sensitive enough to differentiate students' ability on different levels.

**Figure 1:**
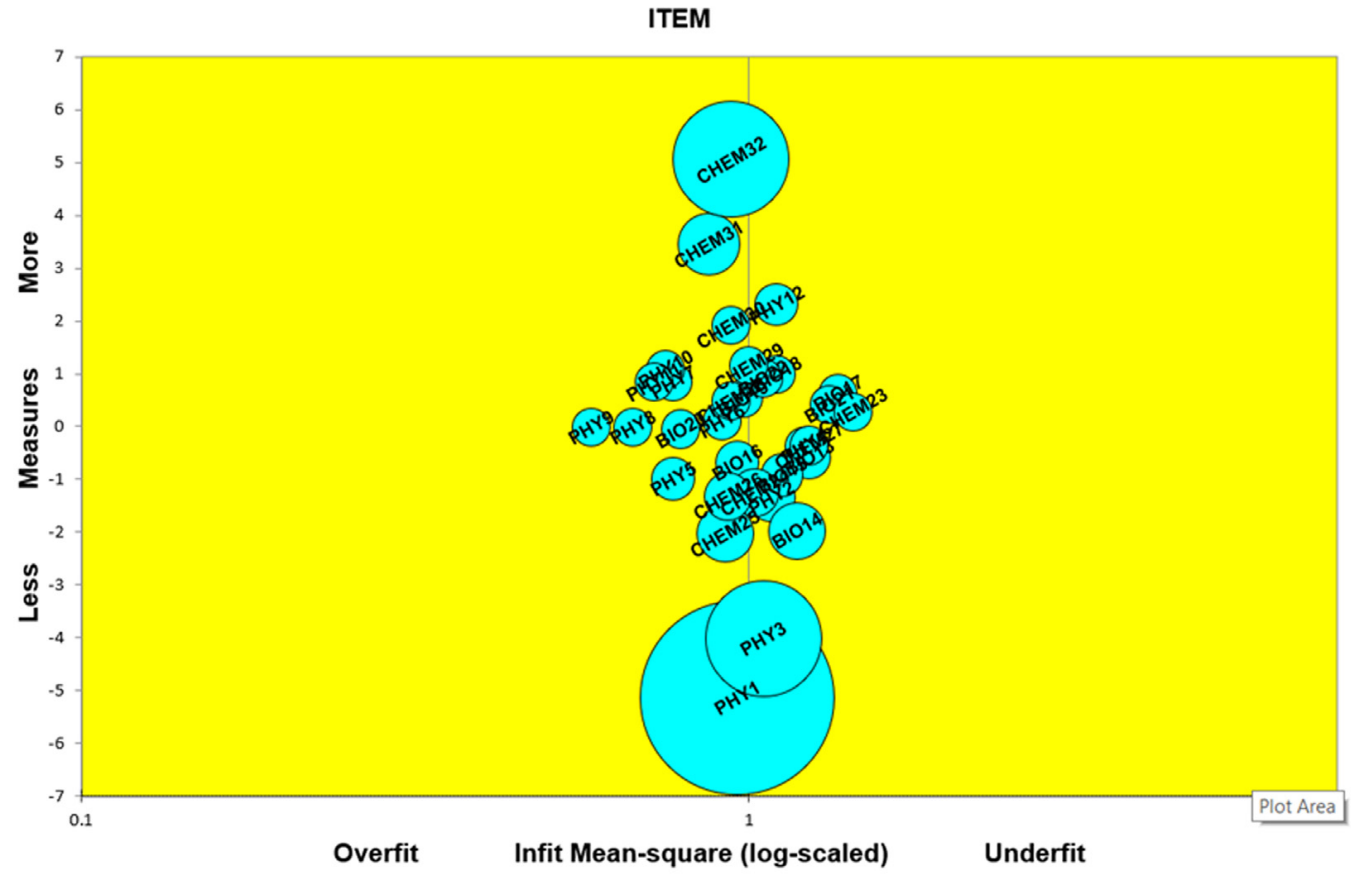
*The bubble chart for item fit criteria based on infit MNSQ*.

Validation criteria based on item fit statistics, standardized mean square residual (ZSTD), and the mean square residual (MNSQ) indicated two items with positive point biserial correlations (PTMA) values: BIO21 (.17) and CHEM23 (.08) do not meet the fit criteria with an outfit MNSQ above 1.6. The ideal outfit and infit MNSQ are 1 based on the Rasch measurement model, but the acceptable values range from 0.5 to 1.5 (approximately 1.6 still acceptable) and infit and outfit ZSTD ranging from −2 to +2 sequentially (Andrich, 2018; Bond et al., 2020). If the MNSQ parameters are acceptable, then ZSTD can be ignored (Linacre‬, 2021b). All items have a positive PTMA, which shows that all items contribute to measuring the differences in students’ abilities at various levels. We, thus, decided to include all items in the analysis. Figure 1 presents item fit criteria based on infit MNSQ.‬‬‬‬‬‬‬‬‬‬‬‬‬‬‬‬‬‬‬‬‬‬‬‬‬‬‬‬‬‬‬‬‬‬‬‬‬

For the person fit criteria, the mean of outfit and infit MNSQ are 0.95 and 1.01, which is close to the ideal threshold around 1, and the mean of infit and outfit ZSTD are -0.1 and 0.1, which are still acceptable. The result from the person fit criteria confirms that participants in this study are fit based on Rasch measurement.

### 5.2 Unidimensionality and local independence

The principal component analysis of Rasch (PCAR) was used to evaluate instrument dimensionality. The two-tier multiple-choice diagnostic test was used to assess student misconception in science, so we assumed that the unidimensionality criteria as a single factor to measure misconception in science as a latent construct. Based on PCAR, a test only measures a dimension if the minimum variance explained by the measure is >30 % (Linacre, 1998). Results showed that the variance explained by measures was 38.5%, showing that the developed test met the unidimensionality assumption.

Local independence confirms that the performance of one item is independent of the performance of other items, with the raw residual correlation between pairs of the items <0.3 (Boone et al., 2013). the items in the test have a residual correlation around 0.1 and 0.28 which means that the assumption of local independence was meet in this study.

### 5.3 Item difficulty pattern between science concepts and disciplines

We calculated the standard deviation (SD) and the mean of average item difficulty measure for each of the three science disciplines, that is, physics, biology, and chemistry, using item difficulty estimates or logits of items (Table 4). Table 4 shows that the mean of items in chemistry was the most difficult than the mean of items in physics and biology. The mean of items in biology was placed as the easiest on the basis of the mean of item difficulties.

**Table 4:** Standard deviation and mean of item difficulty based on the science discipline.

Science discipline	Number of items	Difficulty	
		M	SD
Physics	12	−0.56	2.12
Biology	10	−0.07	0.95
Chemistry	10	0.74	2.23

**Figure 2:**
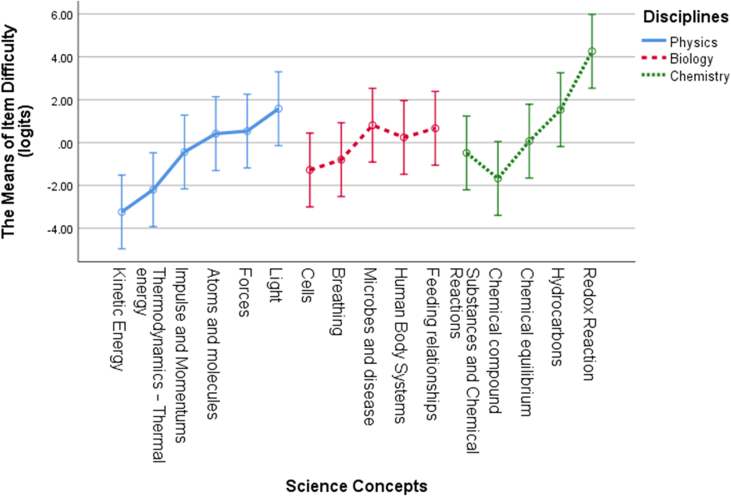
Item difficulty patterns between science concepts and across science disciplines.

Additionally, we also calculated the item difficulty estimates (measure) on the basis of the 16 science concepts as shown in Table 5 in this study. When comparing item difficulty for each concept, the redox reaction (CHEM 32) with 5.06 logits was the most challenging item to solve among all of the items in chemistry, and kinetic energy (PHY1) with −5.13 logits was the easiest item among all of the items in physics. We explore the specific item difficulty estimates for each item number and item fit parameters in Table 5. Figure 2 also represents the item difficulty pattern in specific science concepts to make it easier to understand data distributions of item difficulty levels between the science concepts and the science disciplines.

**Table 5:** Item difficulty estimates and item fit parameters.

Item code	Discipline	Science Concept	Measure (logits)	INFIT MNSQ	OUTFIT MNSQ	PTMA	Source referenced
PHY1	Physics	Kinetic energy	−5.13	0.96	0.13	0.22	(AAAS, 2012)
PHY2		Kinetic energy	−1.35	1.08	1.06	0.37	Authors
PHY3		Thermodynamics—thermal energy	−4.02	1.05	0.43	0.23	Authors
PHY4		Thermodynamics—thermal energy	−0.38	1.21	1.43	0.28	Authors
PHY5		Impulse and momentums	−0.99	0.77	0.61	0.63	Authors
PHY6		Impulse and momentums	0.11	0.91	0.92	0.52	Authors
PHY7		Atoms and molecules	0.84	0.77	0.71	0.61	(AAAS, 2012)
PHY8		Atoms and molecules	−0.01	0.67	0.59	0.72	Authors
PHY9		Force	−0.02	0.58	0.51	0.78	(AAAS, 2012)
PHY10		Force	1.09	0.75	0.65	0.62	Authors
PHY11		Light	0.85	0.72	0.63	0.66	(Csapó, 1998)
PHY12		Light	2.31	1.10	1.14	0.23	Authors

BIO13	Biology	Cells	−0.59	1.23	1.38	0.27	(AAAS, 2012)
BIO14		Cells	−1.97	1.18	0.66	0.36	Authors
BIO15		Breathing	−0.92	1.12	1.52	0.33	(AAAS, 2012)
BIO16		Breathing	−0.68	0.96	1.27	0.44	Authors
BIO17		Microbes and disease	0.63	1.36	1.34	0.16	(AAAS, 2012)
BIO18		Microbes and disease	0.99	1.10	1.06	0.34	Authors
BIO19		Human body systems	0.53	0.98	1.00	0.45	Authors
BIO20		Human body systems	−0.05	0.79	0.71	0.63	Authors
BIO21		Feeding relationships	0.42	1.32	1.72	0.17	Authors
BIO22		Feeding relationships	0.91	1.05	1.02	0.38	(Csapó, 1998)

CHEM23	Chemistry	Substances and chemical reactions	0.28	1.43	1.68	0.08	(AAAS, 2012)
CHEM24		Substances and chemical reactions	−1.25	1.02	0.92	0.43	Authors
CHEM25		Chemical compound	−2.03	0.92	1.25	0.37	Authors
CHEM26		Chemical compound	−1.32	0.93	0.87	0.48	Authors
CHEM27		Chemical equilibrium	−0.36	1.23	1.47	0.26	Authors
CHEM28		Chemical equilibrium	0.49	0.94	1.00	0.48	Authors
CHEM29		Hydrocarbons	1.15	1.00	0.97	0.41	(AAAS, 2012)
CHEM30		Hydrocarbons	1.92	0.94	0.79	0.41	Authors
CHEM31		Redox reaction	3.46	0.87	0.71	0.31	Authors
CHEM32		Redox reaction	5.06	0.94	0.32	0.20	Authors

A two-way Analysis of Variance (ANOVA) was used to analyze the effect of science concepts and science discipline on item difficulty estimates based on logits. The 2 × 2 ANOVA group in this study achieved the assumption of homogeneity variances based on Levene's test (p > 0.05). To validate the normality data assumption, the Kolmogorov–Smirnov test was run before conducting the two-way ANOVA. The results showed that the item difficulty estimates did not differ significantly from normality (p > 0.05) with kurtosis (2.21) and skewness (−0.14).

As presented in Table 6, the results showed a significant effect of science concepts on item difficulty estimates with a large effect size, F (13) = 4.76, p < 0.0. Also, the interaction effect of science disciplines and science concepts showed a significant effect on item difficulty estimates F (15) = 4.59, p < 0.0. However, the difference of item difficulties estimates among science disciplines was found to be insignificant, F (2) = 1.30, p > 0.05. We can assume that there were no significant differences in the population average among the three different science disciplines, i.e., physics, biology, and chemistry, based on a two-way ANOVA, although the difference in the mean logits of item difficulty as shown in Table 4, positioning items in chemistry as being more difficult than items in physics and biology. Both the science concepts and science disciplines can explain 81% of the variance on item difficulty estimates. To sum up, these findings indicated that the item difficulties pattern varies across science concepts, although there are no significant mean differences of item difficulties among disciplines.

**Table 6:** Two-way ANOVA for item difficulty measure.

Dependent variable	Sum of squares	*df*	Mean square	*f*	*p*
Disciplines	9.27	2	4.63	1.30	0.28
Science concepts	81.66	13	6.28	4.76	0.00
Disciplines ∗ Science concepts	90.93	15	6.06	4.59	0.00
R^2^ =.81 (adjusted R^2^ =.63)					

### 5.4 Specific investigation on item difficulty pattern among science concepts

**Figure 3:**
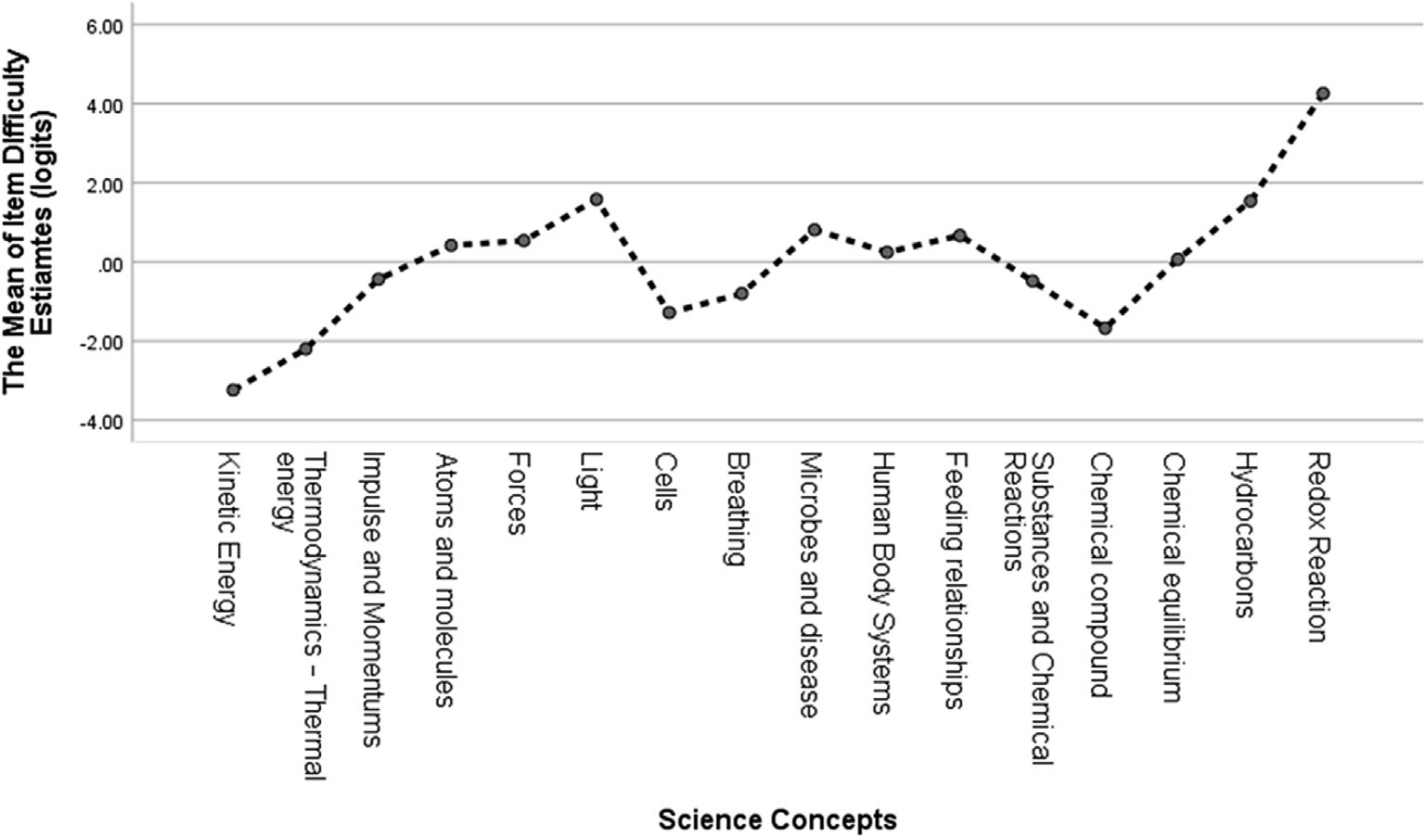
The mean of item difficulty estimates based on science concepts.

For understanding concepts in science distributing misconception to students, we can inspect the item difficulty estimates results from Table 5. The item difficulty estimates can be segmented into four categories; very easy (logits < −1), easy (−1 ≤ logits <0), difficult (0 ≤ logits <1), and very difficult (logits ≥1) (Sumintono and Widhiarso, 2014). Item difficulty estimates in physics showed that concepts of light (PHY11 and PHY12) are more difficult than other concepts in that discipline. All items in physics have logits ranging from −5.12 to 2.13 (very difficult). The concept of kinetic energy (PHY1) is the easiest concept to answer because the concept application can be learned easily. In biology, all item logits are ranging from −1.97 to 0.99. Microbes and disease (BIO 18) have 0.99 of logits (difficult) compared with other items in that discipline, indicating that students have suffered misconceptions and difficulty answering correctly, whereas Cells (BIO 14) is the item that is the easiest one to answer correctly with −1.97 logits. Chemistry has the highest difficulty level among the three science disciplines with logits ranging from −2.03 to 5.06. Redox reaction (CHEM32) has 5.06 logits and was found to be the most difficult item to answer, indicating that students suffer severe misconceptions in redox reaction concepts. To visualize the item difficulty pattern from each concept among disciplines, we calculated the mean of item difficulty pattern for each concept in Figure 3.

### 5.5 DIF based on gender and grade

**Figure 4:**
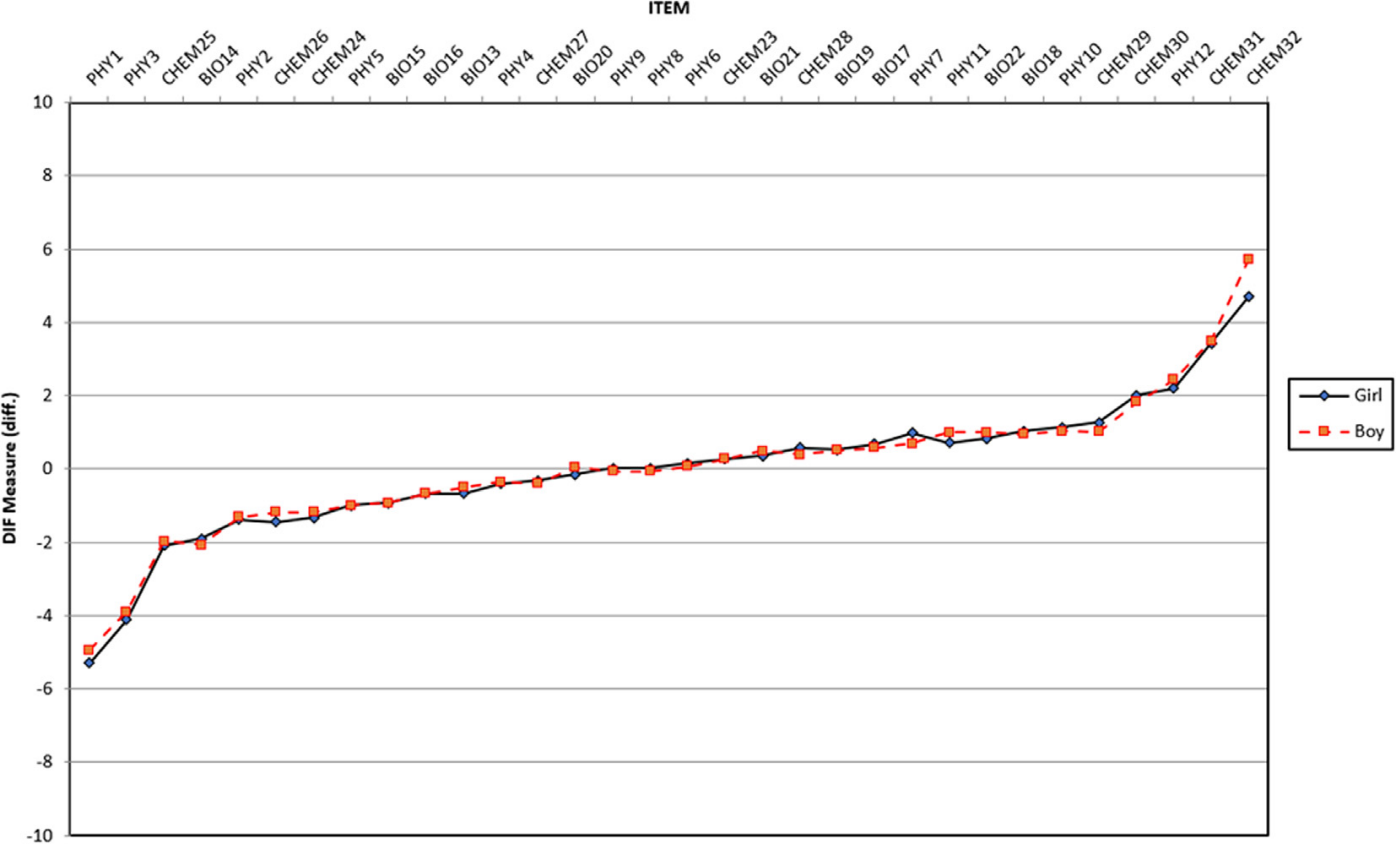
DIF measure based on gender.

**Figure 5:**
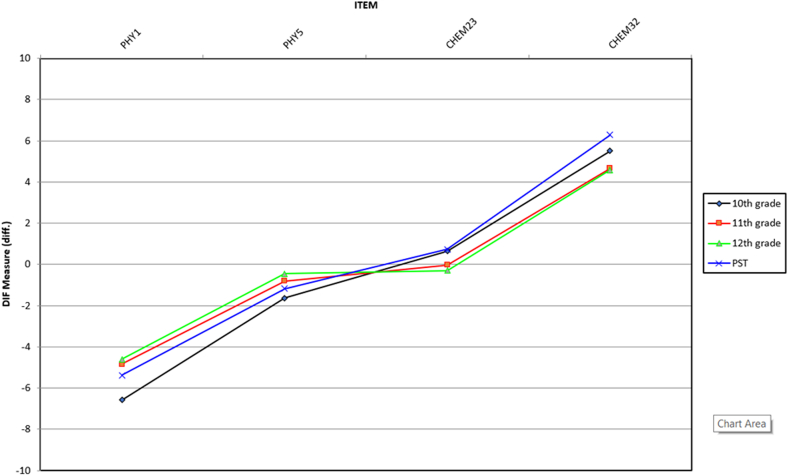
DIF measure based on grade.

DIF analysis was performed to assess differences in item function on the basis of gender and grade on all items in test. DIF analysis investigated item responses on the basis of categorical variables for each item on assessing student misconceptions using a test (Adams et al., 2020; Boone et al., 2013). Differential item functioning analysis is categorized into three types: moderate to large (| DIF | ≥ 0.64 logits), slight to moderate (| DIF | ≥ 0.43 logits), and negligible (Zwick et al., 1999). Figure 4 shows that, overall, items do not have DIF based on gender, except one item in chemistry (CHEM 32). For DIF based on grade, we compared four different cohorts: 10th grade, 11th grade, 12th grade, and the PST. Four items are categorized to differ based on grade: PHY1, PHY5, CHEM23, and CHEM32 (see Figure 5).

## 6 Discussions

Through the statistical analysis, we have confirmed that all items used in the developed instrument meet the valid and reliable criteria according to the parameters for the Rasch measurement. The 32 developed items have outfit and infit MNSQ ranging from 0.13 to 1.72 (see Table 5), whereby ZSTD can be ignored if the sample size is more than 500 respondents (Azizan et al., 2020; Linacre‬, 2021b). Figure 1 shows the item fit pattern based on the MNSQ infit. Several studies had validated the difficulty of items in specific science concepts across science disciplines, such as the concept of energy (e.g., Park and Liu (2019); Neumann et al. (2013)). However, the present study attempts to validate and evaluate item difficulty patterns on various science concepts resulting in student misconceptions that are still limited to the science education area. On the basis of the findings, we can confirm that the item difficulty level is not always reached by students, whereby students must master the more accessible concepts before learning the more complex concepts. This result was in line with previous studies examining the item difficulty level in science subjects (Steedle and Shavelson, 2009), although the science concept under this study is different and the focus is on common concepts causing student misconceptions in science learning.‬‬‬‬‬‬‬‬‬‬‬‬‬‬‬‬‬‬‬‬‬‬‬‬‬‬

The difficulty item pattern in the 16 science concepts studied had different average item difficulty levels based on three specific disciplines offered in Indonesian schools (refer to Table 4). The average value of items in the field of chemistry (M: 0.74 logits, SD: 2.23) was much higher than items in the concept of physics (M: −0.56 logits, SD: 2.12) and biology (M: −0.07 logits, SD: 0.95), whereby items with the redox reaction concept (CHEM32) with 5.06 logits in chemistry are the most difficult items to be understood by students, indicating that students often experience misconceptions of the redox reaction concept. These findings were also supported by previous research by Laliyo et al. (2019) measuring the item difficulty level in the redox reaction concept of 1150 Indonesian students having 1.27 logits with the highest logits measure. This study also assumed that the redox reaction was the concept causing students to experience misconceptions. The concept of the redox reaction is an important topic to understand because the redox reaction helps students understand the phenomena that occur in elements in chemical reactions such as losing and gaining electrons or increasing and decreasing oxidation numbers (Treagust et al., 2014).

The results of the two-way ANOVA show that there is a significant effect on the difficulty estimates of whole items on each science concept, p < 0.05. There is also a significant interaction between science concepts and disciplines. However, the item difficulty estimates did not differ significantly in the three different science disciplines, p > 0.05. These findings are consistent with previous studies that found the item difficulty estimates in science concepts did not differ by science disciplines (Park and Liu, 2019). This finding implies that students’ understanding of various science concepts has a different pattern. However, it tends to be similar across science disciplines, especially in physics, biology, and chemistry, indicating that students have different abilities in solving science problems regarding science concepts.

To investigate the item difficulty estimates for each science concept in the present study, we categorized the average item difficulty estimates for each concept into four categories in Table 7. Four concepts occupy the very difficult categories, namely, forces, light, hydrocarbons, redox reaction. The forces and light concepts in physics subject were also identified as concepts that distribute misconception to students (Kaltakci-Gurel et al., 2017; Soeharto et al., 2019). In chemistry, the hydrocarbons and Redox were also reported as concepts that were difficult to understand, thus causing student misunderstanding in science learning (Erman, 2017; Laliyo et al., 2019; Ramirez et al., 2020). Five concepts are in the difficult category (see Table 7), specifying students’ difficulty in answering or understanding the particular science concept correctly. The item difficulties of each concept were also proven to differ in a previous study by Park and Liu (2019) that reported the item difficulties of the concept of energy concepts in science varied based on students' abilities. Mapping the level of items in science concepts can help teachers realize conditions in teaching specific science concepts considered difficult to learn in classroom activities. By understanding the difficulty level of items in various science concepts, the teacher can estimate which concepts cause students to experience misconceptions in science learning.

**Table 7:** The science concept categorization of item difficulty estimates based on the logits mean.

Very easy (logits < −1)	Easy item (−1 ≤ logits <0)	Difficult item (0 ≤ logits <1)	Very difficult item (logits ≥1)
Kinetic energy, thermodynamics—thermal energy, cells, and chemical compound	Impulse and momentums, breathing, microbes and disease, substances, and chemical reactions	Atoms and molecules, feeding relationships, human body systems, and chemical equilibrium	Force, light, hydrocarbons, and redox reaction

DIF confirms that CHEM32 has differences based on gender. In CHEM 32, the item difficulty estimates for females, DIF measure, is 4.69 logits, and for males, the DIF measure is 5.70. These results were in line with previous studies by (Wyse and Mapuranga, 2009) that reported that DIF might happen based on the respondent background, such as gender, and the DIF measure varies according to the item difficulty level. Hence, the DIF contrast is 1.01 logits indicating females are 1.61 logits less able to address item CHEM 32 than males, so CHEM32 was categorized as moderate to large on DIF. DIF based on grade confirmed that four items were difficult for students to understand based on the school level: PHY1, PHY5, CHEM23, and CHEM32. These findings indicate that the school level or grade has a reasonably significant implication in assessing the differences in students’ ability to work on items on science concepts. Comparing the DIF contrast from 10th grade to 11th grade, 12th grade, and the PST for PHY1, PHY5, and CHEM32, the DIF contrast on PHY1 was categorized into moderate to large DIF with 1.73 logits, 1.99 logits, and 1.28 logits, respectively, showing that students in the 10th grade were less able to solve PHY1 than the other grades. The DIF contrast on PHY5 was categorized into moderate to large DIF with 0.83 logits, 1.18 logits, and 0.46 logits showing students in the 10th grade were less able to solve PHY5 than the other grades. The DIF contrast on CHEM32 was categorized into moderate to large DIF with −0.84 logits, −0.93 logits, and 0.77 logits indicating that students in the 10th grade can better solve item CHEM32 than those in the 11th and 12th grades, but those in the 10th grade have less ability than the PST to solve item CHEM32. The DIF contrast on CHEM23 was categorized into moderate to large DIF for 11th–10th grades (−0.676 logits) and 12th–10th grades (−0.943), the negative values showing that students in the 11th and 12th grades have less ability to solve item CHEM23 than those in the 10th grade.

## 7 Conclusions

In summary, all items in the developed two-tier multiple choices diagnostic test meet the valid and reliable criteria. Our study confirms that the difficulty level of items on various science concepts is not universally based on science topics, but they are connected or similar across science disciplines, especially in physics, biology, and chemistry. We also found particular items in the science concept may have different difficulty levels based on gender and grade.

### 7.1 Limitations and future study

We accept that there are some limitations in our study such as the items not covering all concepts in science learning, only selecting a few items across disciplines that persistently generate misconceptions, the fact that the dataset used only cross-sectional data, and the lack of racking analysis. Some of the limitations outlined above are the improvements that we must consider in further research. Variations of items studied in the further work should be able to cover all science concepts taught across science disciplines, especially at the senior high school level, so that researchers can map the overall item difficulty level of whole science concepts. Time series data collection or longitudinal research design must be added to explore whether there is a change of item difficulty level with the racking method in the Rasch measurement. Racking analysis allows researchers to evaluate whether there is a change in the difficulty level of the item on the different testing times sequentially (Arnold et al., 2018; Linacre‬, 2021b). We feel this research can encourage other researchers to explore further the difficulty level of items in science concepts across disciplines. Understanding the item difficulty level can help teachers be more careful and concerned about conducting learning activities to deliver particular scientific concepts found difficult to comprehend by students.‬‬‬‬‬‬‬‬‬‬‬‬‬‬‬‬‬‬‬‬‬‬‬‬‬‬

For a future study, we will explore in more detail person ability estimates to identify students’ misconceptions and investigate whether students are guessing answers or have inconsistent answer patterns. By evaluating student misconceptions in science, teachers can determine the extent to which students experience misconceptions in science learning.

## Declarations

### Author contribution statement

Soeharto Soeharto and Benő Csapó: Conceived and designed the experiments; Performed the experiments; Analyzed and interpreted the data; Contributed reagents, materials, analysis tools or data; Wrote the paper.

### Funding statement

This work was supported by the 10.13039/501100015763University of Szeged Open Access Fund (grant number: 5436).

### Data availability statement

Data associated with this study has been deposited online at https://figshare.com/articles/dataset/item_difficulites_pattern_sav/16926115/1.

### Declaration of interests statement

The authors declare no conflict of interest.

### Additional information

No additional information is available for this paper.
